# Machine Learning Classification of Inflammatory Bowel Disease in Children Based on a Large Real-World Pediatric Cohort CEDATA-GPGE® Registry

**DOI:** 10.3389/fmed.2021.666190

**Published:** 2021-05-24

**Authors:** Nicolas Schneider, Keywan Sohrabi, Henning Schneider, Klaus-Peter Zimmer, Patrick Fischer, Jan de Laffolie, CEDATA-GPGE Study Group

**Affiliations:** ^1^Institute of Medical Informatics, Justus-Liebig-University Giessen, Gießen, Germany; ^2^Faculty of Health, Technical University of Applied Sciences Mittelhessen, Gießen, Germany; ^3^Department of Pediatrics, Justus-Liebig-University Giessen, Gießen, Germany

**Keywords:** pediatric inflammatory bowel disease, machine learning, diagnostic assistance, convolutional neural network, CEDATA-GPGE registry

## Abstract

**Introduction:** The rising incidence of pediatric inflammatory bowel diseases (PIBD) facilitates the need for new methods of improving diagnosis latency, quality of care and documentation. Machine learning models have shown to be applicable to classifying PIBD when using histological data or extensive serology. This study aims to evaluate the performance of algorithms based on promptly available data more suited to clinical applications.

**Methods:** Data of inflammatory locations of the bowels from initial and follow-up visitations is extracted from the CEDATA-GPGE registry and two follow-up sets are split off containing only input from 2017 and 2018. Pre-processing excludes patients in remission and encodes the categorical data numerically. For classification of PIBD diagnosis, a support vector machine (SVM), a random forest algorithm (RF), extreme gradient boosting (XGBoost), a dense neural network (DNN) and a convolutional neural network (CNN) are employed. As best performer, a convolutional neural network is further improved using grid optimization.

**Results:** The achieved accuracy of the optimized neural network reaches up to 90.57% on data inserted into the registry in 2018. Less performant methods reach 88.78% for the DNN down to 83.94% for the XGBoost. The accuracy of prediction for the 2018 follow-up dataset is higher than those for older datasets. Neural networks yield a higher standard deviation with 3.45 for the CNN compared to 0.83–0.86 of the support vector machine and ensemble methods.

**Discussion:** The displayed accuracy of the convolutional neural network proofs the viability of machine learning classification in PIBD diagnostics using only timely available data.

## Introduction

The incidence and prevalence of Inflammatory Bowel Disease (IBD) have been constantly increasing over the last years, with a relevant rise in Inflammatory Bowel Disease with onset in the pediatric age group (PIBD) (1). A classification of the PIBD at the onset of Crohn's disease (CD), ulcerative colitis (UC) and inflammatory bowel disease unclassified (IBDU) is important to determine treatment and surveillance options during the course of the disease (2). The diagnosis of IBD is based on the Porto criteria through a combination of history and physical exam, stool and lab diagnostic, endoscopic examination and radiological workup and finally histological examination of the gastrointestinal tract (3). Both diseases show some pathognomonic macroscopic appearance, but especially in younger children, the diagnosis can be challenging, since histological appearance in the early stage of the disease may not provide typical morphological changes (4).

To meet the development goals of pediatric patients and avoid poor outcomes, the quality of patient care, diagnosis and treatment should be reconsidered in terms of quality improvement initiatives with the usage of modern information technology (5). This intention motivated the founding of the CED-KQN project, currently supported by German federal innovations funds (VSF17054). CED-KQN is based on the clinical registry CEDATA-GPGE®, in which the disease features of over 5,000 children and adolescents are collected since its founding in 2004. The project members aim to analyze the data of the CEDATA registry, improve patient care and data quality as well as enable self-care in patients and their families (6). Through the usages of Machine Learning and data comparison, predictors of poor outcomes can be identified and decision support for the diagnostic process can be provided.

In this study, the registry data of inflammatory presence of patients during their initial and follow-up examinations is used to create a classification algorithm for the diagnosis of patients, differentiating between CD and UC. Tools for the better classification of diseases are an appealing option for clinicians who aim to optimize diagnosis accuracy and treatment. Preceding research on this subject has shown the possibility of differentiating between CD and UC through the means of Machine Learning techniques (7, 8).

Machine Learning (ML) is a versatile and exceedingly wide science of using algorithms to form determinations or predictions based on past observations, which uses the constantly increasing available computation power to analyze large and/or complex data structures (9). Various ML algorithms use different concepts to identify patterns or to extract features out of given medical data and use these to either classify data or predict outcomes. For example, Yao et al. (10) developed an ML algorithm to predict the outcome of antiepileptic drug treatment based on long-term observation of patients starting from the point of first diagnosis. The risk determination ML algorithm implemented by Weng et al. (11) significantly improves the accuracy of cardiovascular risk prediction relying solely on routine data. This enables early preventive treatment as well as eliminating gratuitous therapy.

Subsequent to the learning or training process, a prediction model can be afterwards analyzed in order to identify contributing or independent parameters and possibly comprehend correlations between data and outcomes (12). While unsupervised ML enables the self-grouping of data into previously unknown categories defined by the algorithm itself, supervised Machine Learning enables the classifying of datasets, which classes or categories are known from the beginning. The supervised model learns from data classified by experts and gains the ability to predict the class of dataset with an unseen or undefined class (13).

This study uses various models for supervised learning, to classify the diagnosis of patients with PIBD. It utilizes the data of the common blood markers CRP (C reactive protein) and ESR (erythrocyte sedimentation rate) in addition to the macroscopic data collected during endoscopic procedures. Data taken from the CEDATA registry along with the corresponding diagnosis was used to train the supervised models. The models were then compared and a convolutional neural network, having achieved the comparably highest accuracy, was chosen for further optimization. The neural network was subsequently improved using grid parameter optimization and it was validated against registry data, not used in the initial training or testing. Our goal is to assert the feasibility of using common clinical data taken from the CEDATA registry to model Machine Learning decision support for diagnosis.

## Materials and Methods

### Ethics

Data of all patients collected in the CEDATA registry since 2004 was initially included. All patients were below the age of 18 years. CEDATA-GPGE® is a prospective, multicenter registry for PIBD in German-speaking Countries (14). It is approved by ethic committees of all participating institutions. Written informed consent to participate in the registry is provided by the patients' legal guardians. Further ethical approval or consent was not required for this study as the research is covered by the CEDATA-GPGE®'s ethical approval and the consent of its participating patients.

### Data-Extraction and Pre-processing

The registry currently contains records of over 45,000 visitations where the patient suffers from either CD or UC. This yields an overall ratio of 2.01 observations with a CD diagnosis to one with a UC diagnosis. The extent of inflammation is assessed upon initial diagnosis and during the course of disease. The location features in the registry describe the presence of inflammation from the esophagus to the rectum. The 11 locations from the esophagus to the rectum, collected in CEDATA, include the esophagus, stomach, duodenum, ileum, terminal ileum, coecum, ascending colon, transverse colon, descending colon, sigmoid and rectum. After the identification of the described features and additional laboratory and administrative information, an anonymized dataset was exported from the database of the CEDATA registry.

The extracted dataset was filtered to remove patients in remission at the time of examination to avoid negative impact on the learning effect of the algorithms. Incomplete documentations, with no information in any of the features of interest, were removed from the data to achieve a higher quality of training and test data sets (15). All visitations were then categorized as either a follow-up visitation or an initial specialist contact and the data set was split accordingly. From each visit of both datasets, the location data was extracted as well as the diagnosis. In addition, the CRP and ESR laboratory results were added to the follow-up data. The macroscopic findings were encoded in numerical values [0, 1]. A visible presence of inflammation is represented as “+1” while the unaffected gastrointestinal wall was coded as “0.” In the dataset containing follow-up visitations, the CRP results were coded as “mg/dl” unit and the unit of ESR as “mm/h.” These laboratory values were, respectively, normalized to a decimal zero to one, to better suit the processing by Machine Learning algorithms (16). Two subsets of data were formed for the follow-up visitations, consisting only of information entered into the registry in the years 2017 and 2018, respectively. These were chosen to analyze the effect recent data quality improvement measures had on the prediction performance. All datasets resulting from the pre-processed information were consecutively split into a training and a test set. The former is employed for training, including cross-validation and hyperparameter tuning, while the latter is used in the final performance evaluation. The ratio of observations in the training and validation set is 70 to 30% of the whole dataset (17).

### Machine Learning Methods

With the aim of finding the best-suited method for classification, a support vector machine (SVM), Random Forrest (RF), extreme Gradient Boosting (XGBoost) as well as dense and convolutional neural networks algorithms (DNN, CNN) were trained and validated on two different datasets derived from the training dataset (18). The initial learning and evaluation process employs the model's default configuration values, adjusting only necessary input and output values as needed for the prescribed use case. To avoid overfitting, a 10-fold cross-validation was implemented. This repeats the process of training the model multiple times while selecting different, non-overlapping, equally proportioned subsets of data from the training dataset. The first and larger one is used for training, while the second is used for validation (19). This usage of additional data not present in training sets also assures a better performance of models on external data (20). The performance of each algorithm was assessed by the calculated average accuracy of the model after all iterations of the cross-validation. For further optimization and the implementation of a highly efficient model, the convolutional neural network was chosen.

The applied neural net consists of a convolutional layer, a pooling layer and a fully connected layer. Through an artificial network, features of the given data are extracted and weighed individually. The information is then pooled to condense and reduce it. Afterward, the concluding fully connected layer handles the final classification of the given input (21).

### CNN Optimization

For optimization of the convolutional neural network, several hyperparameters are of varying importance, influencing not only accuracy but also the computational efficiency of training a model. Both, convolutional and fully connected layer, have activation functions which can be changed. These functions are attached to each neuron of the layer and determine whether the neuron should be activated for each input (22). For the neural net, there are also different optimizers and loss functions available. The loss functions are used to evaluate the wrongness of the predictions of the network. This information is then used by the optimizer to adjust the weights of the model. Additionally, modern optimizers attempt to improve the training by estimating best weight changes based on the previous weight values. Furthermore, the number of epochs, defining the quantity of training iterations, as well as the batch size, specifying the quantity of the individual observations included in a training process, can be optimized (23). The improvement of hyperparameters is dependent on the task of the model and the data and thus the influence of a parameter on the accuracy of the model cannot be predicted at the start of implementation The best hyperparameters were chosen using a grid-search-algorithm in combination with five-fold cross-validation. In this process a matrix is constructed, containing all possible parameter combinations from the previously composed lists. Afterward, the cross-validation process including training and testing the model is carried out for each of the matrix's cells, comparing all accuracies to find the optimal combination (24). The process of optimization was carried out for each of the follow-up datasets and the initial contact dataset individually.

After identifying the optimal parameters of the convolutional neural network, the resulting model was again trained using 10-fold cross-validation by employing the combined data of the training and test datasets. Its performance was analyzed regarding mean accuracy and standard deviation, as with the previous algorithms. The process was repeated for all the datasets, including a visitation set without laboratory values. Following the analysis and model training for all datasets, prediction models were received for the entire follow-up data including and excluding laboratory values, only visitations entered in 2018 or 2017 and the entire initial contacts.

Extraction of data from the CEDATA registry was completed using a self-written Ruby script (2.5, Japan, Yukihiro Matsumoto). The pre-processing of data, as well as model implementation, was accomplished with Python (3.7, USA, Python Software Foundation) and its packages Pandas (0.24.3, Community-developed), Keras (2.2.4, USA, François Chollet), and Scikit-learn (0.21.2, France, Community-developed).

## Results

**Figure 1:**
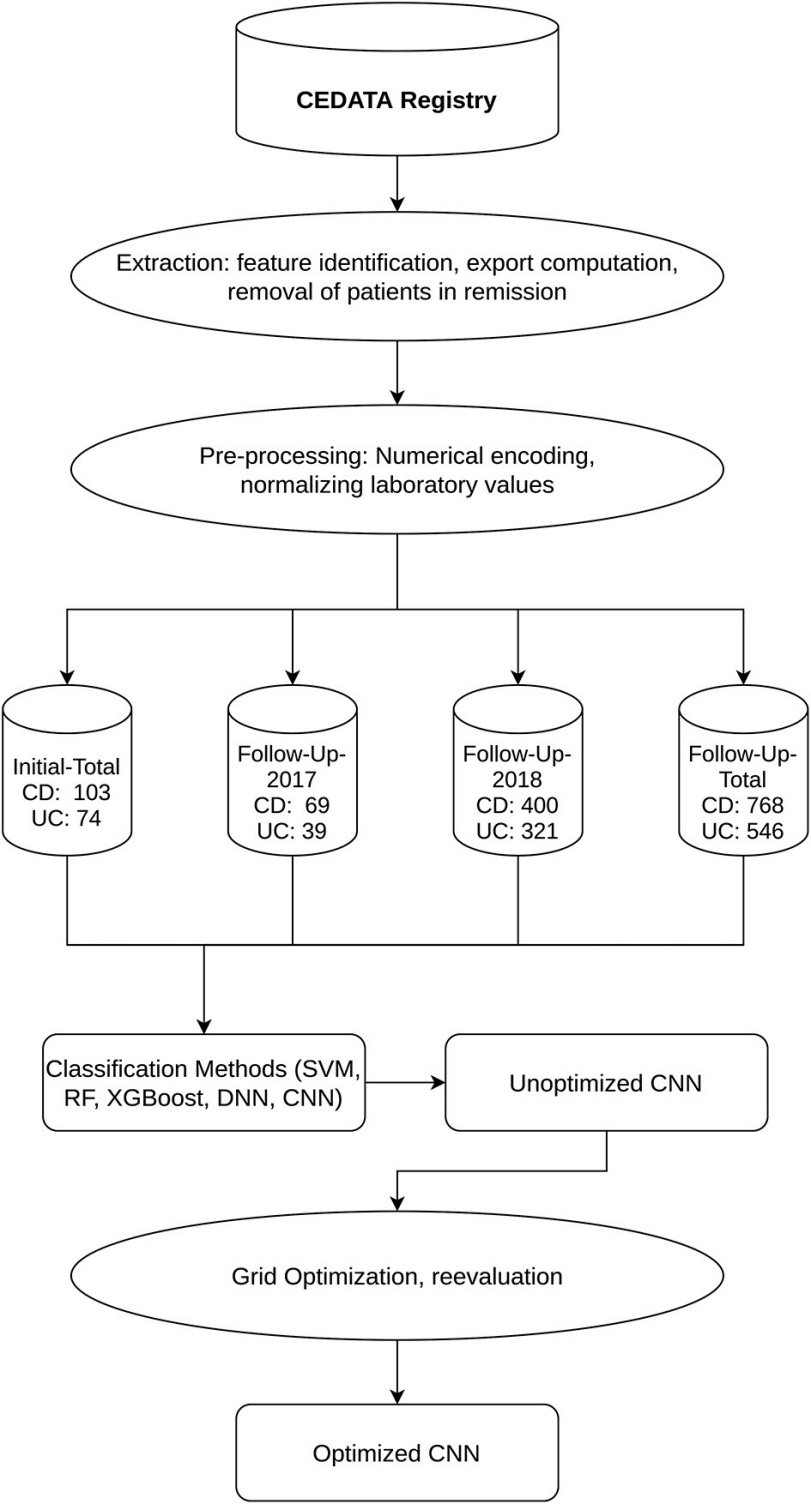
Workflow and Datasets displays the outline of the applied methodology as well as the resulting datasets and their segmentation into CD and UC diagnosis.

**Figure 2:**
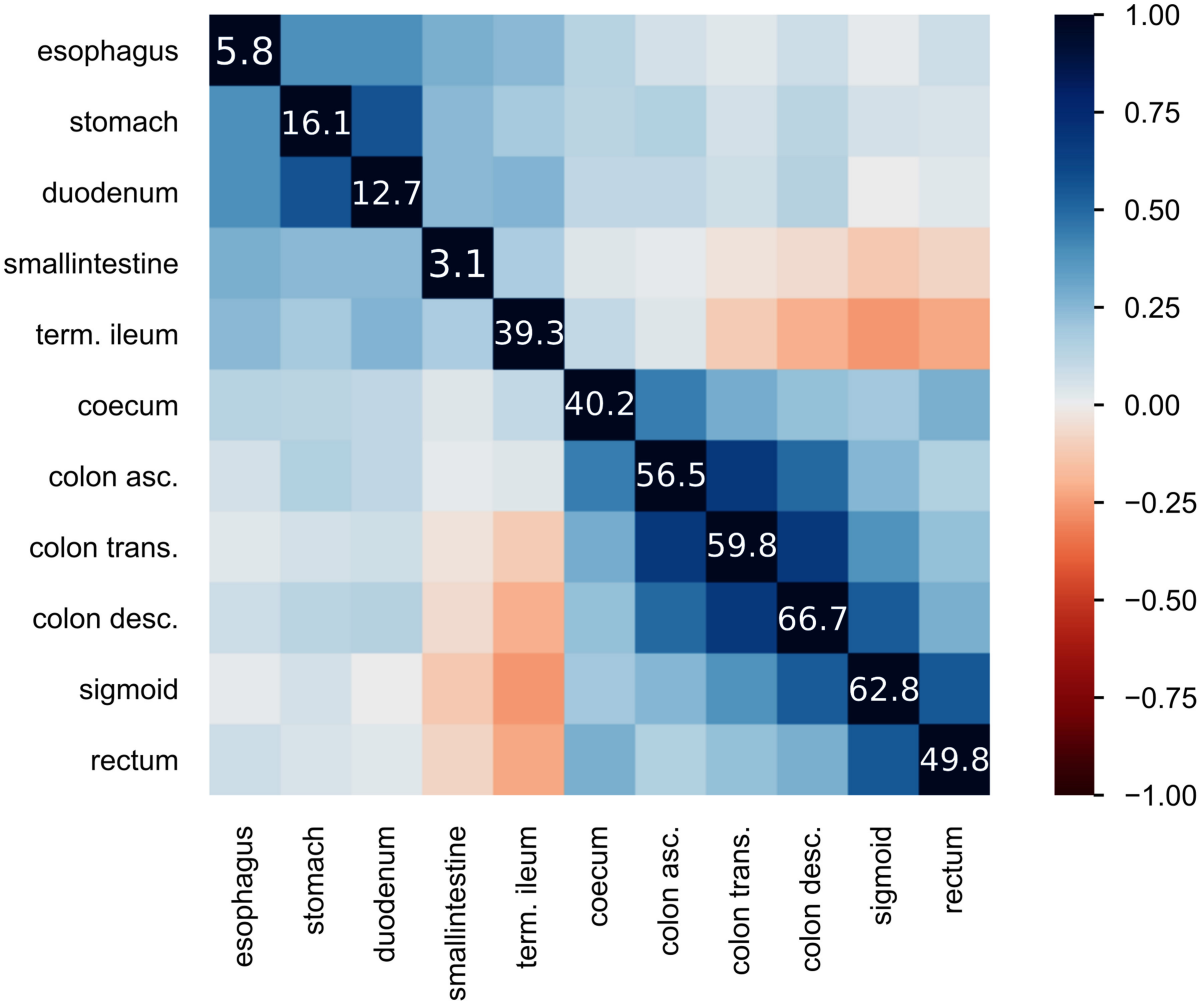
Heatmap of inflammatory locations**:** displays the correlation of observed inflammations in the given locations. Correlation is visualized using Pearson's “r” coefficient. Additionally, the diagonal line of values shows the frequency of inflammation observed at the location in percent.

The CEDATA registry contained records of 29,556 follow-up visitations where the patient suffers from CD and 14,394 from UC at the time of data extraction. Comparatively, there are 3,135 recorded initial contacts where the patient suffers from CD and 1,837 in which the diagnosis is UC. The four different datasets derived from the database are composed of up to 1,314 visits for the entire follow-up visitation set (“Follow-Up-Total”), 768 of which derive from cases the patient was inflicted by CD and 546 cases in which they were suffering from UC. The data limited to follow-ups from 2018 (“Follow-Up-2018”) consist of 721 entries, among them 400 with a CD diagnosis. The smaller datasets for 2017's follow-up examinations (“Follow-Up-2017”) and the initial documentations (“Initial-Total”) amount to 108 and 177 records, respectively. The diagnosis count and ratio of each dataset are displayed in Figure 1. The patient group consists of 44.7% females. The frequency of inflammation at the observed locations is highest in the descending colon with 66.7%, followed by the sigmoid with 62.8%. An inflammation of the small intestine is least frequently recorded at 3.1%. The incidence at each location can be viewed in Figure 2, which also displays the correlation between inflammations at each location. While neighboring positions generally show a high correlation, inflammations in both the small intestine and terminal ileum show a contrary relationship with inflammation of the colon and only weakly correlate with each other or the coecum. Various machine learning algorithms were used on each visitation. The span of the accuracy of classifying between CD and UC range from 83.49 to 90.57% on the Follow-Up-2018 dataset with extreme gradient boosting showing to be least accurate. Following XGBoost, is the support vector machine and the other ensemble method random forest which performed better reaching an accuracy of 85.49 and 86.53%, respectively. The most efficient models are all different artificial neural networks (NN), with naive implementations starting at 88.78% and reaching 90.02% through the not optimized convolutional neural network on the 2018 follow-up dataset. After an optimization process, this method reached 90.57% on the same dataset. The choice of the convolutional neural network for further optimization was based on the performance of the different classification strategies as shown in Table 1. The SVM and both ensemble methods result in a significantly lower standard deviation at 0.83 and 0.87 than the utilized neural networks where the lowest is 3.45 through the optimized convolutional neural network. The naive convolutional implementation and the dense neural network yield 4.01 and 5.43 each.

**Table 1:** Achieved accuracy per method for 2018 follow-up dataset: table showing the executed machine learning methods and the achieved accuracy in percent as well as standard deviation during cross validation.

**Machine learning method**	**Accuracy in percent (%)**	**Standard deviation**
SVM	85.49	0.83
RF	86.53	0.87
XGBoost	83.94	0.87
Dense NN	88.78	5.43
Conv. NN	90.02	4.01
Conv. NN optimized	90.57	3.45

*Of the naive implementations, the convolutional neural network perfomes best at 90.02% which is topped by its optimized implementation reaching 90.57 percent. The underliing data stems from registry inputs of 2018 and includes laboratory values*.

**Figure 3:**
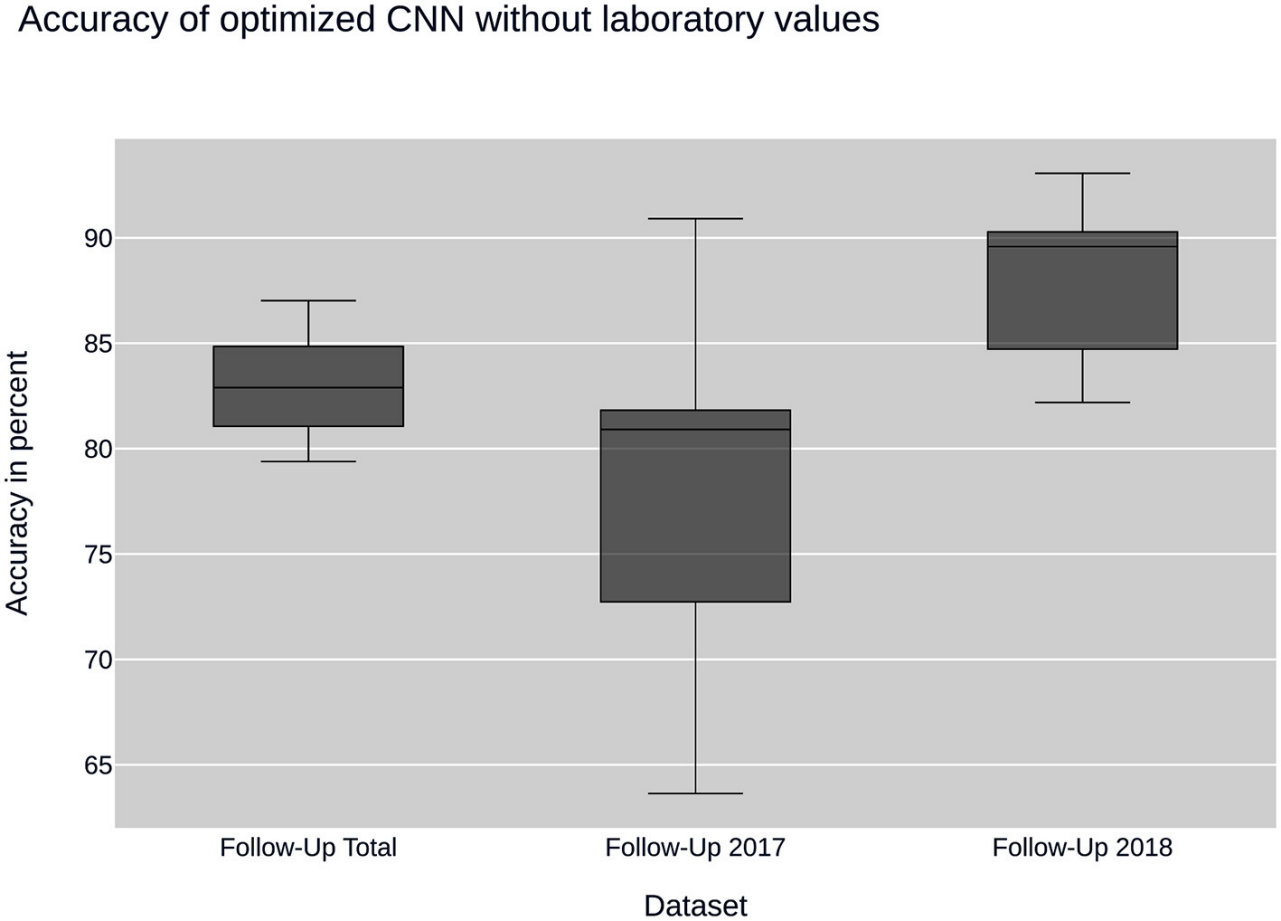
CNN performance without laboratory values: plots the performance of the convolutional neural network (CNN) when using sub datasets selected by time of input. None of the datasets contain laboratory values.

**Figure 4:**
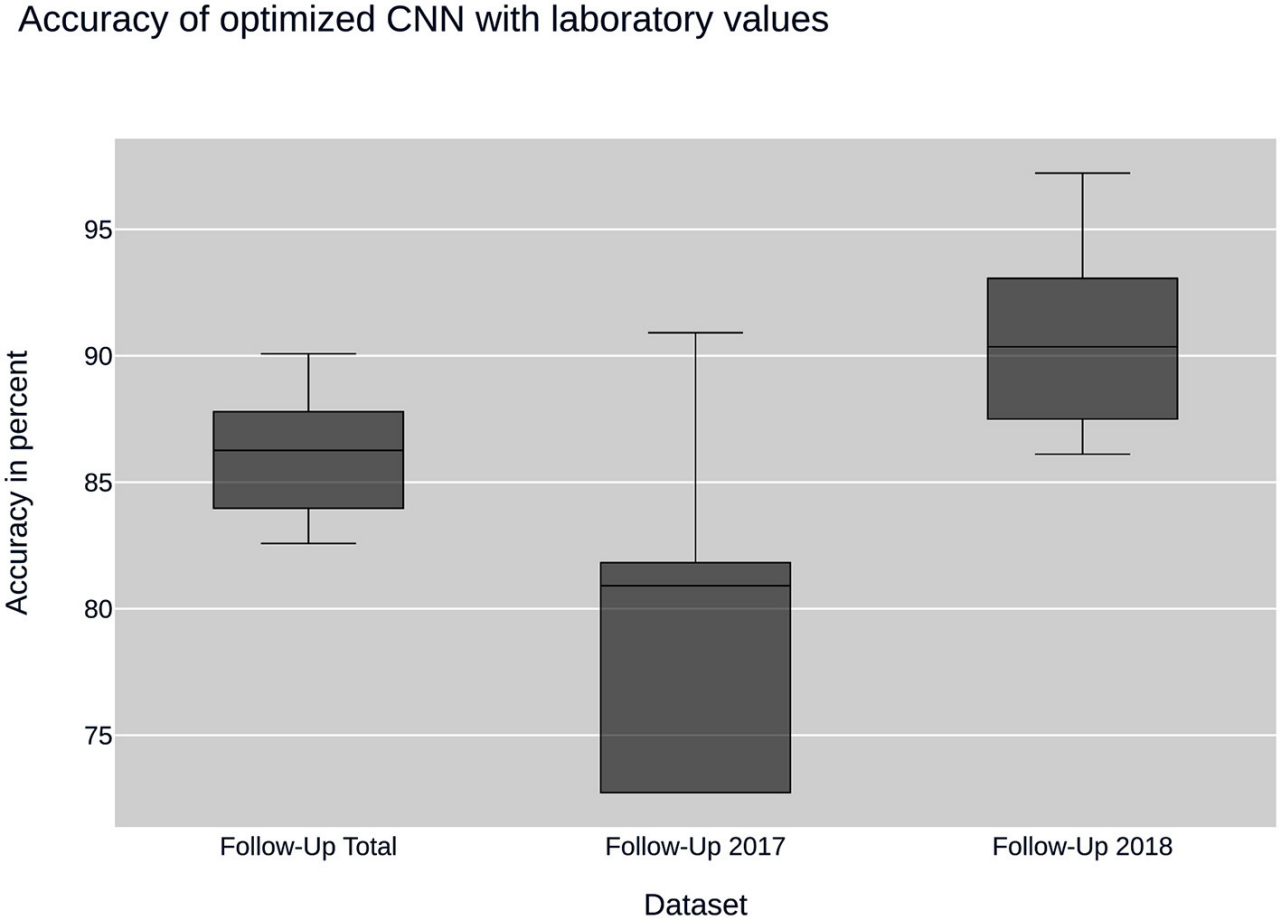
CNN performance with laboratory values: illustrates the performance of the convolutional neural network (CNN) on datasets containing laboratory values. The datasets are sampled by year of collection.

Further insight into classification was gained using the convolutional neural network. Ancillary datasets were used during the training and evaluation process to explore the influence of common laboratory parameters and to examine the influence of changes in data acquisition by the CEDATA registry software over the past years. Figure 3 shows the accuracy achieved by the optimized convolutional neural network using the data subsets containing no laboratory values. In addition, the models differ in the time span, in which their data was recorded. The first model was trained on the Follow-Up-Total dataset, while the second and third only contain data registered in the years 2017 and 2018 (Follow-Up-2017, Follow-Up-2018), respectively. While the whole dataset results in an accuracy of 83.25%, the data from 2017 equates to 78.81%. The subset containing visitations input in 2018 shows an efficiency of 88.21%. In comparison, Figure 4 displays the resulting accuracy when splitting the dataset by the input timespan and including laboratory parameters. The entire dataset results in 86.15% accuracy, showing an improvement of 2.9% when compared to the original result from the optimized neural network trained on the dataset without laboratory values. The model trained on the dataset comprised of entries from 2017 achieves an enhanced outcome with an accuracy of 79.72%. The model trained and validated on records collected in 2018 demonstrates the best performance at an accuracy of 90.57%, an increase of 2.36% compared to the model of the same dataset excluding laboratory values. The classification models for the Initial-Total dataset seen in Table 2 reached an accuracy between 82.89 and 87.06%. The performance increase of the optimized CNN is 0.12% when compared to the not optimized model of the same dataset.

**Table 2:** Achieved accuracy per method for initial visitation dataset: table showing the executed ML-methods and the achieved accuracy on Initial-Total dataset in percent.

**Machine learning method**	**Accuracy in percent (%)**
SVM	85.89
RF	83.33
XGBoost	82.89
Dense NN	86.89
Conv. NN	86.94
Conv. NN optimized	87.06

*Of the naive implementations, the convolutional neural network perfomes best at 86.94% which is topped by its optimized implementation reaching 87.06%. The underliing data includes laboratory values*.

The optimized neural networks were compiled with the following values: the activation function selected for the convolutional layer is the hyperbolic tangent function. The fully connected layer implements the Rectified Linear Unit function. As loss function, the mean squared error function was found to achieve best results. The corresponding optimizer is “Adam” (derived from adaptive moment estimation). The grid search for optimal hyperparameters resulted in a batch size of 64 and a number of epochs of 200 for the follow-up visitations and 100 epochs and 128 batch size for the initial contacts.

Overall, the neural nets trained and tested on data acquired in 2018 show a better performance than the ones used on data from 2017, which themselves have a disadvantage over data taken from the entire dataset. The precision and recall as well as the F1 score show a similar behavior, displaying peak values on data from 2018 and worst on the 2017 dataset.

The addition of common laboratory values has a positive effect on the model's performance, with effects varying from an additional 2.42 to 3.19% compared to the accuracy without using laboratory parameters. The neural network has a higher sensitivity than specificity in perspective to Crohn's disease resulting from a larger number of visitations falsely classified as CD while in fact representing cases of ulcerative colitis. Being a two-class classification problem, the sensitivity and specificity are reversed for UC as can be observed in Table 3.

**Table 3:** Sensitivity and specificity of CNN per diagnosis: this table displays the sensitivity and specificity of the convolutional neural network (CNN) trained and tested on the data from the Follow-Up-2018 dataset.

**Disease**	**Sensitivity (in %)**	**Specificity (in %)**
Crohn's disease	91.4	89.73
Ulcerative cloitis	89.73	91.4

During further research, the remaining not optimized machine learning methods were optimized as well. This process did not yield any further insights and the results are not shown but can be forwarded by the authors on request.

## Discussion

This study, based on the CEDATA registry and its collected information, displays the optimization of an algorithmic model for supporting decision-making when classifying PIDB patients concerning their diagnosis. The results show the possibility of digital differentiation between CD and UC with an accuracy of over 90% (90.57%), despite the intersection features visible during endoscopic procedures and the presence of not more than common laboratory values.

Plevy et al. (8) demonstrated a method for achieving solid accuracy on the classification of IBD using Machine Learning methods on serologic markers in addition to macroscopic data. The resulting model is albeit not applicable to average patient data as the used markers are expensive and generally not obtained in real-world hospitals. Hence, these markers are not included in the clinical registry CEDATA either. Mossotto et al. (7) aimed to provide a Machine Learning model based on simpler clinical data like microscopic and macroscopic examination results and still achieve good classification accuracy. In another approach, Dhaliwal et al. (25) also utilized histological findings to classify PIBD subgroups on a small cohort. Building on this approach, commonly available clinical data is used in the CEDATA registry study to classify CD and UC through Machine Learning algorithms, waiving histological data as well as any other momentary data of the illness' presentation. Following the same reasoning, the timely progress of the disease is also excluded, keeping requirements for utilizing the model low. It should also be noted that the histological results are highly dependent on the examiner and the technical equipment (26). The limited data requirements enable the application of the model on cases of PIBD where either histological data or extensive serology reports are not yet on hand or are not conclusive. The resulting model is applicable to PIBD patients only due to significant differences in inflammation localization. The differences in adult and pediatric IBD localization (27) proves that machine learning results, stemming from adult data, cannot be assimilated into pediatric models and signifies the importance of purely pediatric classification models as well as distinct data collection for research purposes (27). Although cross-validation mitigates the possibility of overfitting, it results in fewer data to train a classifier (20). Large data sources like the CEDATA registry are of high value when trying to apply machine learning methods to medical data and are needed to develop predictors more applicable to real-world scenarios than models fitted on small datasets (25). While the applied pooling and cross-validation technics reduce overfitting, a remaining standard deviation of 3.45 can indicate that the CNN still experiences overfitting during the training phase. To further address this problem, the networks architecture could be expanded, including one or several dropout layers, which randomly drop connections between layers during training and lessen their linkage (28). Other methods might include “early stopping” which prevents further training of the network when the peak performance is reached or “weight decay” which continually decreases the weights of the network during the training phase (29).

A convolutional neural network is more often seen in approaches of image or language recognition, but also applicable for classification (30). While the convolutional neural network is able to classify most data entries correctly, the partial overlap between CD and UC inflammation patterns still leads to misclassifications. The increased performance of the algorithm when applied together with laboratory values points to a close connection of systemic inflammation with IBD diagnosis. The clearest distinction of IBD utilizing the locations visibly inflamed can be made on the initial diagnosis, as there is no corruption through various therapy methods. As the visitations used in the described study took place during ongoing therapy and regular examinations, the noted localizations are expected to be influenced by previously mentioned therapy. Overlap between the macroscopical manifestations has increased when compared to information derived from untreated patients. A convolutional neural network trained on the information of the initial endoscopic diagnostic could be expected to provide better accuracy due to a lower treatment bias on newly diagnosed patients when compared to follow-up examinations. However, the models trained on initial contact datasets consistently performed slightly worse than those fitted with follow-up data. This could be caused by bias in the data due to unrecorded treatment prior to consulting a specialist or the smaller amount of data from initial visitations available in the registry. Another explanation can be found in the missing laboratory values in the initial contact dataset, which were shown to improve model performance in the follow-up dataset. At the time of the creation of the CEDATA registry, the chosen laboratory values of CRP and EST were not included in the data gathered at the initial specialist visitation. Data quality in these fields compromised usefulness in the model on initial presentation. While improving the predictive accuracy of the convolutional network, the hyperparameters were tuned using a grid search algorithm that chose the best parameter combination available by iterating through all given possibilities and saving the one yielding the best result. This approach is as exhaustive as the lists of parameters given to the algorithm. Since there was only a limited list of numbers given as possible values to a number of epochs and batch size, the carried-out grid search is not fully exhaustive. While this approach diminishes computational load and leads to a faster discovery of optimal parameters, the possibility of a better performing combination of hyperparameters remains. To achieve selecting the best combination of elements for the optimization, an exhaustive grid search including all possible epochs and batch sizes would have to be applied. As this process would be incredibly resource-demanding while probably not yielding better results, it is not considered best practice. The performed grid search or even a random search over the given parameters are considered the better approaches to hyperparameter tuning (31).

The differentiation between multiple datasets by date of acquisition was chosen regarding the changes the CEDATA registry and its essential software components underwent through the past years. As all data gathering and entering are prone to errors, mistakes cannot be ruled out among the datasets exported from the registry. This has been the key motivation to continuously improve the data quality and insertion methods used for the storage. The main efforts began with a new user interface at the end of 2017, providing auto-completion, feedbacks and logical assessments, ruling out many potential error sources. The improving performance of the neural network on datasets from 2017 and 2018 is an indicator that these efforts are effective and lead to an overall better data quality. The higher accuracy on the entire dataset compared to 2017 can be attributed to the inclusion of the 2018 data and the overall amount of data provided to the machine learning algorithm. Nevertheless, the whole data from the registry is expected to have high data quality, as patients are continuously examined, their progression closely monitored and decisions like diagnosis are ascertained by multiple physicians.

The CEDATA registry is continuously expanding its storage, not only adding new patients but also joining new data fields to the existing ones. This results in a repository of over 600 possible attributes per visitation. The latter can enable the researcher in further improving the neural network based on the inclusion of additional phenotyping attributes, which might be key to reaching a higher accuracy and could help to identify key parameters when differentiating between PIDB diagnoses. Another possibility would be the inclusion of microbiome omics data. Kellermayer et al. (32) have shown the correlation between this data and an inflammatory process in the intestines. The usage of this data could increase accuracy but also identify specific parameters that have an especially strong correlation to differing PIBD diagnosis.

To further progress the research on this subject, a combination of the different implemented machine learning models is planned. The goal is to utilize the strengths of the individual methods to attain a superior model. A simple path to achieve this would be the calculation of a weighted mean across the incorporated methods. While this does in fact utilize the output of all models, it disregards the fact the models might be more or less accurate for a specific subgroup of samples. Therefore, a method that incorporates differently weighted methods for particular data records is necessary. In regards to this, further research will apply sample-specific late fusion methods to this classification problem (33).

As all data used in this study stems from the CEDATA registry and its associated clinics, there is an underlying bias in the data acquisition and all results should be confirmed with data collected from a non-CEDATA member registry or clinic. To assert if the registries data can accurately represent real-world data, a test with an external institution is planned. Next to validating the model on outside data, the goal is to affirm a high correlation between the registry and overall real clinical data.

The ground truth against which all models were trained and tested is recorded as a diagnosis in the registry. It is noteworthy that physicians formed these diagnoses upon reviewing the entire medical information of the patient and not only the location data. There is no estimate, at what accuracy an experienced medical practitioner might classify the given data, so a direct comparison between the model and a human is impossible. The pre-existent demand for implementing machine learning technics in clinical environments is furthered by the increased attention new deep learning methods have gained in the last years. Rising computational resources and improved models enable novel strategies to support diagnosis, outcome and risk prediction, as well as personalized therapy. However, applying artificial intelligence in medicine should aim not to replace patient-physician interactions and decision processes, but to support them through assistance. As the prediction methods are limited by the entered data, they are unable to regard the patients in their entirety. Even if sufficient data for classification is present, medical experience and individual case assessment are essential for uncommon pathological presentations. Although artificial neural networks are trying to replicate the learning progress of biological brains on a crude level, there is no method to simulate complex pattern recognition and decision process formed through prolonged clinical occupation. Deep Learning algorithms are influenced strongly by the selected training cohort, which can lead to bias of ethnicity and unexpected performance drops when applied on fringe groups (34, 35). Still, various examples show the potential gain of introducing machine learning into clinical processes. Exemplary, there are systems for risk evaluation, pre-processing or pre-sorting of telemetric and visual data, differentiation of patients into subgroups or integration into diagnostic procedures. Overall, the application of artificial intelligence in medicine is beneficial but requires ethical oversight in implementation and usage (36).

The benefit provided by the developed model can be utilized to a great extent immediately in the CEDATA registry itself. Through direct feedback on data input, the physician can receive a probability value for the patient's diagnosis, which can lead to a re-evaluation of the diagnosis or a correction of the recorded data. Thereby, the overall data quality of the register can be improved and an additional care quality assertion for partaking patients is established. The machine learning algorithm itself can be implemented to perpetually learn from the newly input data and continue to increase in accuracy.

An accuracy of up to 90.57% is achieved when differentiating between the diagnosis of CD and UC on relapsed pediatric IBD patients by adapting a convolutional neural network. While this performance is sufficient for usage in the CEDATA registry, an augmentation to at least 95% accuracy seems necessary to implement the model directly in a clinical environment. To achieve this result, further optimization, more data entries and the inclusion of additional visitation parameters into the model will be utilized. The introduction of the commonly examined laboratory parameters CRP and ESR results in a more accurate prediction by the model. It performs better when being tested and validated only with data that has been through a correction and validation process which provides an improved data quality. These results stress the role of high quality and quantity input data to improve decision-support solutions.

## Data Availability Statement

The datasets presented in this article are not readily available because the CEDATA-GPGE registry as well its contained data are owned by the registered association “CEDATA-GPGE®”. Therefore, the propagation of data originating from the register can only be approved by the owner. Requests to access the datasets should be directed to https://www.gpge.eu/cedata-gpge, https://www.gpge.eu/s/1809_projektantrag_cedata-gpge.docx.

## Ethics Statement

CEDATA-GPGE® is a prospective, multicenter registry for PIBD in German speaking Countries (14). It is approved by ethic committees of all participating institutions. Written informed consent to participate in the registry is provided by the patients' legal guardians. Further ethical approval or consent was not required for this study as the research is covered by the CEDATA-GPGE®'s ethical approval and the consent of its participating patients.

## Author Contributions

NS carried out the model performance-analysis, implemented the models, co-wrote the manuscript with JL and designed the required toolchain. KS conceived model comparison and visualization as well as the infrastructure for data extraction. HS contributed data analysis and extraction tools. K-PZ coordinated data collection and research. PF devised algorithm optimization methods with NS and supported neural network design. JL designed the research plan, managed data acquisition, co-wrote the manuscript with NS and headed the interdisciplinary exchange. The CEDATA-GPGE-study-group participated in patient recruitment for this analysis. All authors contributed to the article and approved the submitted version.

## Acknowledgments

We thank all patients and their families for their support.

### CEDATA-GPGE Study Group Members

Rüdiger Adam (Mannheim, Germany), Antje Ballauff (Krefeld, Germany), Barbara Baumgartner (Zwettl, Austria), Thomas Berger (Datteln, Germany), Jens Berrang (Dortmund, Germany), Edith Bieck (Torgau, Germany), Guido Bürk (Herne, Germany), Andreas Busch (Tübingen, Germany), Martin Claßen (Bremen, Germany), Söhnke Dammann (Ravensburg, Germany), Christian Dörfler (Erfurt, Germany), Gesche Düker (Bonn, Germany), Fokko Elschner (Augsburg, Germany), Axel Enninger (Stuttgart, Germany), Annette Findeisen (Greifswald, Germany), Gunter Flemming (Leipzig, Germany), Dirk Föll (Münster, Germany), Marcus Franzke (Salzgitter, Germany), Michael Friedt (Düsseldorf, Germany), Ulrich Gabel (Oberursel, Germany), Rainer Ganschow (Hamburg, Germany), Stephan Gaupp (Nürnberg, Germany), Lars Geerdts (Cottbus, Germany), Johann Gerein (Frankfurt, Germany), Patrick Gerner (Essen, Germany), Alexandra Glettler (Steyr, Austria), Katrin Gröger (Wurzen, Germany), Wiebke Hachmann (Mönchengladbach, Germany), Ralf Hanusch (Rodewisch, Germany), Almuthe Hauer (Graz, Austria), Matthias Heiduk (Plauen, Germany), Peter Heinz-Erian (Innsbruck Austria), Georg Heubner (Dresden, Germany), Monika Hofmann (Chemnitz, Germany), Axel Hübeler (Aue, Germany), Hassan Issa (Leisnig, Germany), Simone Jedwilayties (Friedrichshafen, Germany), Petra Jesche (Hoyerswerda, Germany), Wolfgang Kamin (Hamm, Germany), Hea-Sook Kim-Berger (Marburg, Germany), Peter Klipstein (Quedlinburg, Germany), Ute Kloß (Berlin, Germany), Henrik Köhler (Erlangen, Germany), Andreas Krahl (Darmstadt, Germany), Benno Kretzschmar (Eisenach, Germany), Monika Kurzai (Jena, Germany), Martin Laaß (Dresden, Germany), Thomas Lang (Starnberg, Germany), Ralph Melchior (Kassel, Germany), Michael Melter (Regensburg, Germany), Andreas Möckel (Borna, Germany), Dietrich Ney (Hamburg, Germany), Ralf Pallacks (Memmingen, Germany), Kai Nils Pargac (Riesa, Germany), Maike Petersen (Heidelberg, Germany), Edmund Petri (Münster, Germany), Carsten Posovszky (Ulm, Germany), Markus Prenninger (Wels, Austria), Michael Radke (Potsdam, Germany), Olaf Raecke (Esslingen, Germany), Stefan Rauschenfels (Braunschweig, Germany), Heike Reck (Zittau, Germany), Thomas Richter (Leipzig, Germany), Burkhard Rodeck (Osnabrück, Germany), Frank Schmidt (Halle, Germany), Anjona Schmidt-Choudhury (Bochum, Germany), Heike Schoen (Breitenbrunn OT Erlabrunn, Germany), Thomas Scholbach (Chemnitz, Germany), Christina Schwerk (Leipzig, Germany), Wolfgang Sperl (Salzburg, Austria), Armin Stach (Leverkusen, Germany), Edith Sterniczky (Oberwart, Austria), Thomas Stuckert (Zwickau, Germany), Andreas Vécsei (Wien, Austria), Christoph von Buch (Bad Kreuznach, Germany), Tobias Wenzl (Aachen, Germany), Ahlke Willenborg (Berlin, Germany), Ulf Winkler (Bautzen, Germany), Stefan Wirth (Wuppertal, Germany), Harald Andrew Zaunschirm (Krems, Austria), Bernd Zimmer (Rüsselsheim, Germany).

## Conflict of Interest

The authors declare that the research was conducted in the absence of any commercial or financial relationships that could be construed as a potential conflict of interest.
